# Validity and reliability of the Musicians’ Health Literacy Questionnaire, MHL-Q19

**DOI:** 10.3389/fpsyg.2022.886815

**Published:** 2022-09-23

**Authors:** Christine Guptill, Teri Slade, Vera Baadjou, Mary Roduta Roberts, Rae de Lisle, Jane Ginsborg, Bridget Rennie-Salonen, Bronwen Jane Ackermann, Peter Visentin, Suzanne Wijsman

**Affiliations:** ^1^School of Rehabilitation Sciences, University of Ottawa, Ottawa, ON, Canada; ^2^Faculty of Rehabilitation Medicine, University of Alberta, Edmonton, AB, Canada; ^3^Department of Rehabilitation Medicine, School for Public Health and Primary Care, Maastricht University, Maastricht, Netherlands; ^4^School of Music, University of Auckland, Auckland, New Zealand; ^5^Centre for Music Performance Research, Royal Northern College of Music, Manchester, United Kingdom; ^6^Africa Open Institute for Music, Research and Innovation, Stellenbosch University, Stellenbosch, South Africa; ^7^School of Medical Sciences, Faculty of Medicine and Health, University of Sydney, Sydney, NSW, Australia; ^8^Department of Music, University of Lethbridge, Lethbridge, AB, Canada; ^9^Conservatorium of Music, University of Western Australia, Perth, WA, Australia

**Keywords:** health literacy, musicians’ health, occupational health, validity, reliability, psychometrics

## Abstract

High prevalence of musicians’ physical and mental performance-related health issues (PRHI) has been demonstrated over the last 30 years. To address this, health promotion strategies have been implemented at some post-secondary music institutions around the world, yet the high prevalence of PRHI has persisted. In 2018, an international group of researchers formed the Musicians’ Health Literacy Consortium to determine how best to decrease PRHI, and to examine the relationship between PRHI and health literacy. An outcome of the Consortium was the development of a new health literacy tool for musicians, the MHL-Q19, which drew from the theoretical framework of the European health literacy suite of tools, HLS-EU. The aim of the current study was to evaluate the validity and reliability of the MHL-Q19. Participants completed a battery of questionnaires, including the HLS-EU-Q16 for the assessment of general health literacy; the Musculoskeletal Pain Intensity and Interference Questionnaire for Musicians (MPIIQM); the RAND-12 quality of life questionnaire; and the General Self-Efficacy scale (GSE). We hypothesized that the MHL-Q19 would have a weak correlation with the HLS-EU-Q16; moderate correlation with the physical component scale and weak correlation with the mental component scale of the RAND-12; moderate correlation with the GSE; and finally, moderate correlation with pain interference and weak correlation with pain intensity of the MPIIQM. A total of 549 post-secondary music students from six English-speaking countries completed the battery of questionnaires, and 328 of these participants provided valid responses to the MHL-Q19 alone 2 weeks later. The tool showed acceptable internal consistency and test–retest reliability. Three of our hypotheses were supported, although the strength of the correlations varied from what we had predicted. The fourth hypothesis was not supported; our findings indicate that lower health literacy scores were weakly related to higher MPIIQM pain intensity and interference scores. The results of this study support the notion that musicians’ health literacy is a distinct construct that cannot be fully evaluated with existing health literacy tools. Given that this is a new instrument, the evidence presented is positive and promising. Further studies will be needed to refine the tool.

## Introduction

Over 30 years of research has demonstrated that many musicians around the world experience significant performance-related physical and mental health conditions, including repetitive strain; peripheral neuropathy; depression; and generalized and performance anxiety (Fishbein et al., 1988; Zaza, 1998; Guptill et al., 2000; Bragge et al., 2006; Paarup et al., 2011; Ackermann et al., 2012, 2014; Kenny and Ackermann, 2015; Steinmetz et al., 2015; Baadjou et al., 2016; Kenny and Asher, 2017; Fernholz et al., 2019; Stanhope et al., 2019). Poor health-promoting behaviors have long been evident in musicians (Williamon and Thompson, 2006; Kreutz et al., 2008; Ginsborg et al., 2009; Kenny and Ackermann, 2016), and specific risk factors for musicians have been identified, both intrinsic and extrinsic, including social, organizational and cultural factors (Chan and Ackermann, 2014; Vaag et al., 2014; Araújo et al., 2017; Perkins et al., 2017; Waters, 2019, 2020). Health promotion strategies have been initiated, mainly in post-secondary music education programs, but health education is not always successful in changing musicians’ health behaviors (Spahn et al., 2017; Baadjou et al., 2018; Matei et al., 2018; Norton, 2020; Baadjou et al., 2021).

The importance of health literacy for improving personal health behaviors and community health has been recognized by the World Health Organization (WHO) and in public health research (WHO, 1998; see also Nutbeam, 1998; Okan et al., 2019). Sørensen et al. (2012) define health literacy as follows: “Health literacy is linked to literacy and entails people’s knowledge, motivation and competences to access, understand, appraise, and apply health information in order to make judgments and take decisions in everyday life concerning healthcare, disease prevention and health promotion to maintain or improve quality of life during the life course.” (p.3). Research has increasingly recognized the importance of context and relational aspects in determining individuals’ health literacy (McKenna et al., 2017; Geboers et al., 2018; Sørensen et al., 2021). Occupational health literacy models that account for the environmental and social determinants of health in workplaces have been developed (Rauscher and Myers, 2014; Jørgensen and Larsen, 2019), as have population-specific health literacy tools designed to address the variability of these contextual and relational aspects in different occupational settings (Shannon and Parker, 2020; Suthakorn et al., 2020).

In 2018, the Musicians Health Literacy Consortium (MHLC) was formed, bringing together a panel of international experts in musicians’ health. Our intention was to provide a global perspective on how health education can address the persistence of performance-related health issues (PRHI) among musicians, and the role health literacy may play in influencing their health behaviors (Baadjou et al., 2019). An outcome of the MHLC collaboration was the development of an occupational health literacy tool specifically for musicians to measure their abilities to access, understand, appraise, and apply health information concerning their performance health, the Musicians’ Health Literacy Questionnaire (MHL-Q19) (Wijsman et al., forthcoming).^1^

The aim of the current study was to evaluate the validity and reliability of the MHL-Q19 among post-secondary music students.

## Materials and methods

### The research team

The research team included tertiary music educators RL, BR-S, PV, SW and health professionals specializing in musicians’ health, CG, VB, BA, all of whom, like JG, are academic researchers in musicians’ health; a senior graduate student research coordinator with expertise in music education and musicians’ health research, TS; and an academic researcher with expertise in applied measurement, MRR.

### Developing the items

**Figure 1:**
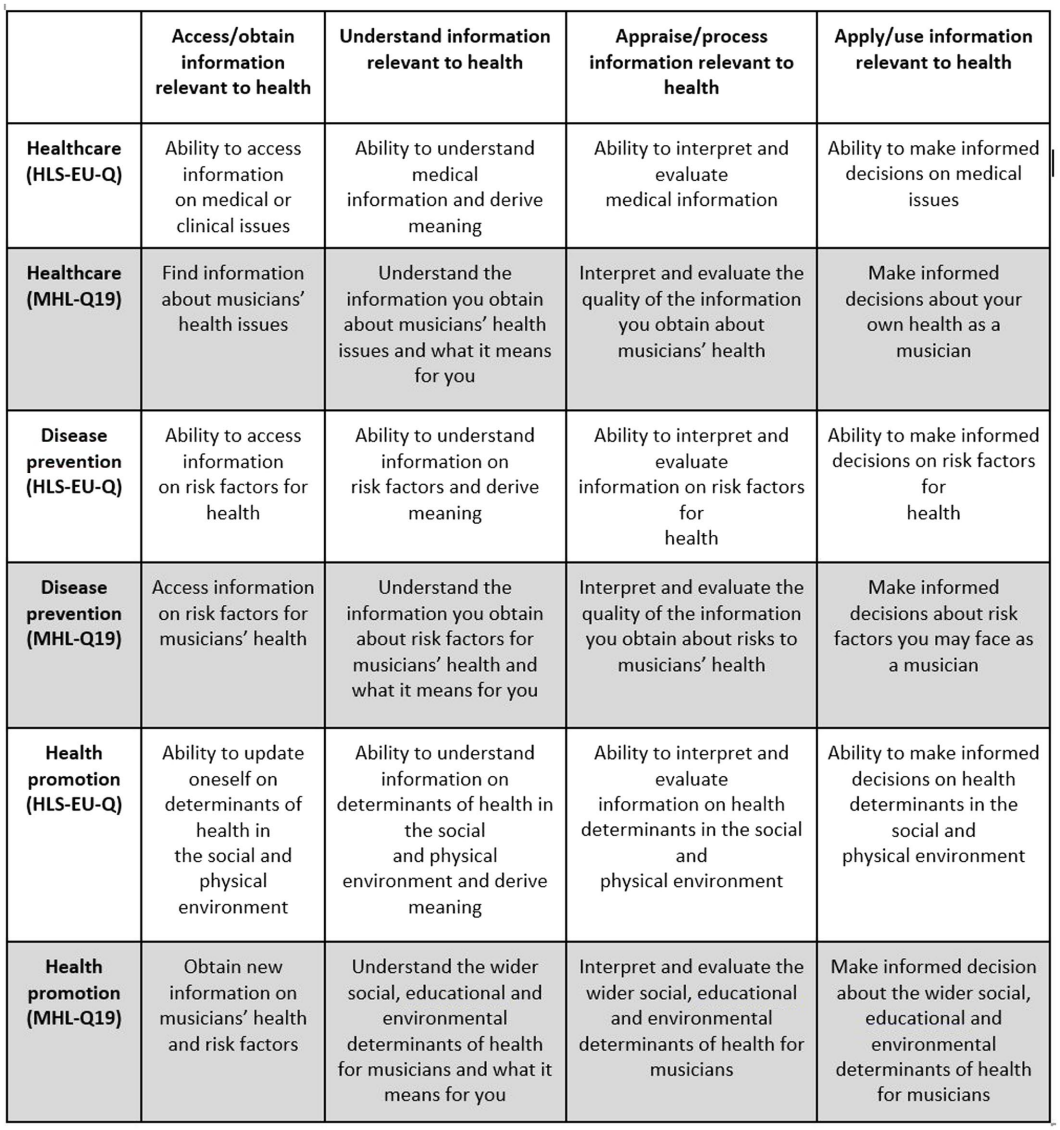
Four dimensions of health literacy across three domains of health: original HLS-EU health literacy matrix (Sørensen et al., 2012; used with permission) and adapted MHL-Q19 matrix (Wijsman et al., forthcoming; see footnote 1).

**Figure 2:**
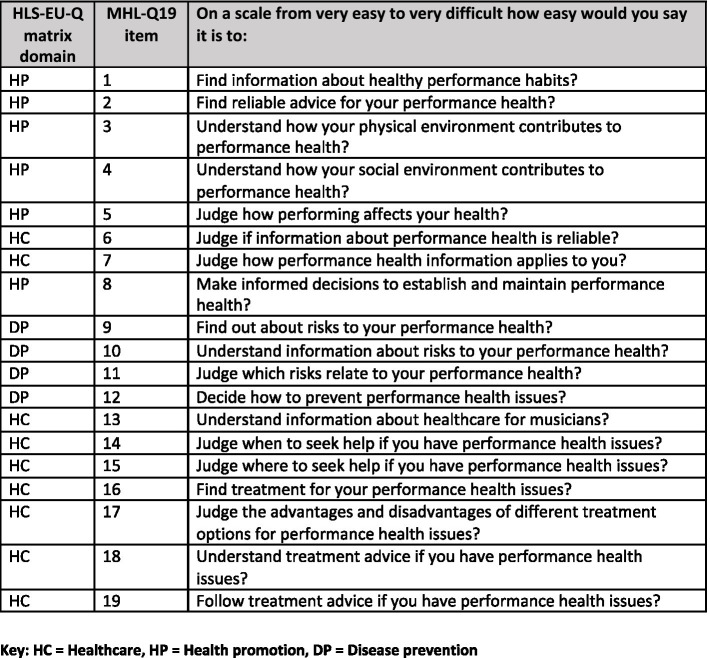
Questions from the Musicians’ Health Literacy Questionnaire, MHL-Q19, and their corresponding HLS-EU-Q matrix domains.

The MHL-Q19 was modeled on the theoretical framework and health literacy matrix of the European Health Literacy Survey, the HLS-EU-Q (Sørensen et al., 2012, 2013, 2015), designed to measure health literacy, in relation to general health, in European populations. The HLS-EU health literacy matrix outlines the interaction of four dimensions of health literacy (accessing, understanding, appraising and applying health information) with three domains of health (Healthcare, Disease Prevention and Health Promotion; Sørensen et al., 2012; Pelikan et al., 2019; see Figures 1, 2). These three domains can be further understood through their relationship with the four dimensions of health in the theoretical framework (Figure 1). In other words, the Healthcare domain can be understood as a person’s ability to access, understand, appraise or interpret, and apply information relating to medical or clinical issues and advice. The Disease Prevention domain is concerned with accessing, understanding, appraising or interpreting, and applying information on risk factors for health. Finally, the Health Promotion domain is concerned with accessing, understanding, appraising and applying information related to determinants of health (Sørensen et al., 2012).

The HLS-EU-Q framework was chosen as a model, first, because it incorporates the assessment of health literacy competencies discussed in the published research literature on musicians’ health behaviors that the team deemed essential to musicians’ health literacy: decision-making, evaluation, responsibility, and self-efficacy (Daykin, 2005; Williamon and Thompson, 2006; Kreutz et al., 2008; Ginsborg et al., 2009; Araújo et al., 2017). Second, the HLS-EU-Q was designed for multi-lingual administration; therefore, deriving items for the MHL-Q19 from the HLS-EU-Q enhances the ability to adapt our new tool for different cultures, which is desirable in a tool designed for use with the global population of musicians.

The MHLC undertook a consensus development process over a 6-month period, adapting the wording of the subscales of HLS-EU matrix to make it more suitable for the musician population, and generating 19 musician-specific items based on questions in the HLS-EU-Q47 (Wijsman et al., forthcoming; see footnote 1). Items specific to musicians’ performance-related health were modeled on questions in the HLS-EU-Q, avoiding the use of medical terminology and focusing on context-specific aspects of musicians’ health literacy. In addition, questions were worded to be inclusive of singers (in future; the current sample consisted of instrumental musicians only); to be inclusive of all musical genres; and to be easily translated into other languages. For example, Question 17 of the HLS-EU-Q47 reads: “On a scale from very easy to very difficult, how easy would you say it is to: find information about how to manage unhealthy behavior such as smoking, low physical activity and drinking too much?” Question 1 of the MHL-Q19 frames this in terms of musician-specific health concerns, asking: “On a scale from very easy to very difficult, how easy would you say it is to: find information about healthy performance habits?” (Wijsman et al., forthcoming; see footnote 1).

### Face validity

The preliminary version of the tool was distributed to a stakeholder network developed by the lead author. This group was comprised of 12 field experts, including administrators of music programs at three Canadian post-secondary institutions; senior leaders of five national-and provincial-level music organizations; one healthcare practitioner specializing in musicians’ health; and three musicians’ health researchers. These experts assessed the face validity of the questionnaire items and gave open-ended feedback on the content of the tool *via* email and during a teleconference, as described in Wijsman et al. (forthcoming; see footnote 1). Based on the generally positive feedback from the stakeholder network, no changes were made to the tool at this stage.

### Construct validity

To assess the construct validity of the new MHL-Q19, we administered it along with four additional validated questionnaires assessing relevant constructs. We also sought to determine whether there were, indeed, elements of health literacy for musicians that were not sufficiently addressed by the other questionnaire tools. The tools employed were as follows:

- The HLS-EU-Q16 (a shorter form of the HLS-EU-Q47) is a short-form assessment of general health literacy (Pelikan and Ganahl, 2017). This tool consists of 16 questions. The overarching question is: “On a scale from very easy to very difficult, how easy would you say it is to:” with possible answers of *very easy*, *easy*, *difficult*, *very difficult*, and *don’t know*. *Very easy* and *easy* responses are assigned a score of 1, and *difficult* and *very difficult* are assigned a score of 0. *Don’t know* is scored as missing data. Total scores of 0–8 are defined as representing inadequate health literacy, 9–12 problematic, and 13–16 adequate. The tool takes about 3 min to complete. Concurrent validity was confirmed by correlations of 76% with the HLS-EU-Q47 and the Newest Vital Sign test (Pelikan and Ganahl, 2017; Bas-Sarmiento et al., 2020). It also demonstrates high internal consistency (Bas-Sarmiento et al., 2020). Reliability was reported to be high in several studies in which the tool was translated into other languages (Bas-Sarmiento et al., 2020; Niedorys et al., 2020; Nolasco et al., 2020).
- The Musculoskeletal Pain Intensity and Interference Questionnaire for Musicians (MPIIQM; Berque, 2014; Berque et al., 2014; Schmidt, 2017) is one of two validated tools purporting to measure musculoskeletal pain in musicians, the other being the Musculoskeletal Pain Questionnaire for Musicians (MPQM; Lamontagne and Bélanger, 2012). This questionnaire asks participants to map the location of symptoms of playing-related musculoskeletal disorders (based on a definition related to that developed by Zaza (1998). Participants use a Likert-type scale to answer a series of questions aiming to determine their level of pain and the degree to which it interferes with their ability to play music at the level to which they are accustomed. We chose the MPIIQM because, at the time that this study was first proposed, the MPQM had only been validated in French on a sample of 31 professional musicians. The MPIIQM validation, by comparison, was conducted with a sample of 37 musicians in English. The two-factor structure (pain intensity and pain interference) was supported by confirmatory factor analysis. The tool had high internal consistency and good test–retest reliability (Berque, 2014).
- The RAND-12 (Johnson and Maddigan, 2004; Cheak-Zamora et al., 2009) is equivalent to the SF-12, derived from the 116-item Medical Outcomes Study designed to measure physical and mental quality of life (QOL). Both tools have been used for decades in health research around the world and have been shown to have good reliability and validity in adults with musculoskeletal (Mani et al., 2017) and chronic health conditions (Johnson and Maddigan, 2004), and mental illness (Huo et al., 2018). Two summary scores can be calculated using these tools: the physical component score (PCS) and the mental component score (MCS). Unlike the SF-12, the RAND-12 does not require a license or the use of the proprietary scoring algorithm, which assumes that the PCS and MCS are unrelated. Instead, we employed the algorithm developed by Johnson and Maddigan (2004) for use in research on individuals with chronic health conditions.
- The General Self-Efficacy (GSE) scale (Schwarzer and Jerusalem, 1995; Schwarzer, 2014) was selected because the literature indicates that health literacy and self-efficacy are related (Xu et al., 2018; Beasley et al., 2020; Wayment et al., 2020). In addition, we chose a measure of general self-efficacy rather than specific music-related self-efficacy, as we felt that such an item would be more related to efficacy in music performance activities, and less related to health literacy. The GSE was first developed in 1979, and the current version was reduced to 10 items in 1981 (the version used in this study; Schwarzer and Jerusalem, 1995). While information does not appear to be available for psychometrics of this tool in its initial development, subsequent studies have established that the GSE has high internal consistency (*α* = 0.86; Scholz et al., 2002) and good test–retest reliability (ranging from 0.47 to 0.75 in studies cited by Scholz et al., 2002). With respect to validity, the tool performed as expected in relation to Bandura’s theory of behavior change (1997), correlating positively with self-esteem and optimism, and negatively with anxiety, depression and physical symptoms, as would be expected (Schwarzer and Born, 1997; Schwarzer et al., 1999). Finally, the scale was demonstrated to be unidimensional, supporting its use as a single measure of general self-efficacy.

We hypothesized that these constructs would be related to musicians’ health literacy and, therefore, that scores on the MHL-Q19 would be positively correlated with those on the four questionnaires. Our hypotheses were as follows:

1. There would be a weak correlation between MHL-Q19 and HLS-EU-Q16 scores because the MHL-Q19 is designed to measure context-specific health literacy in musicians related to their performance-related health, using questions that are similar to those in the HLS-EU-Q47 from which the HLS-EU-Q16 is derived, but that capture a more specific subset of health literacy concerns not addressed in the general health literacy questionnaire (please see Methods – Developing the Items);
2. There would be a moderate correlation between MHL-Q19 and MPIIQM pain interference scores but a weak correlation between MHL-Q19 and MPIIQM pain intensity scores. While there is insufficient evidence about the relationship between health literacy and pain intensity and interference, in the research team’s expert opinion, informed by combined clinical, pedagogical and applied research experience, musicians whose symptoms interfere with performance are more motivated to seek care; thus, we postulated that they would also be more motivated to seek information, thereby increasing their music performance-specific health literacy;
3. There would be a moderate correlation between total MHL-Q19 scores and the physical component scores, and a weak correlation between MHL-Q19 scores and mental component scores of the RAND-12. This is because the research literature suggests (Douglas, 2019) that information about physical health is more accessible to music students than information about mental health;
4. There would be a moderate correlation between MHL-Q19 and GSE scores because the literature has demonstrated a relationship between health literacy and self-efficacy (Xu et al., 2018; Beasley et al., 2020; Wayment et al., 2020).

### Participants and recruitment

The MHLC panel agreed with other researchers that post-secondary training is an ideal time to deliver health promotion and injury prevention education (Chesky et al., 2006; Ginsborg et al., 2012; Norton, 2016; Salonen, 2018), because music students are at significant risk of PRHI, are highly engaged in educational activities, and are motivated to take actions to safeguard their future careers as musicians. Therefore, this initial validation study was completed with post-secondary music students from 13 institutions worldwide. We excluded singers from the sample, because the MPIIQM has only been validated with instrumental musicians. All recruitment was conducted electronically, by email to students directly and through social media using avenues frequented by post-secondary music students.

To calculate the required sample size, we looked at the total number of music students from available data: in the US, 332,297 post-secondary students in 2011, or ~0.1% of the current US population (College Music Society, 2015), and in Australia, 3,500 students in 2011, or ~0.01% of the current Australian population (Global Access Partners, 2011). We used 0.05% as the estimated percentage of the population in the other countries where data were collected (Canada, New Zealand, South Africa and the United Kingdom), for a total of 421,297 students. The estimated sample size required to achieve 95% confidence with 5% margin of error is 384 students (Qualtrics sample size calculator, https://www.qualtrics.com/blog/calculating-sample-size/).

A total of 549 post-secondary music students completed the battery of questionnaires, including participants from Canada (*n* = 253), the United States (*n* = 138), South Africa (*n* = 43), Australia (*n* = 57), New Zealand (*n* = 31), and the United Kingdom (*n* = 27). See Table 1 for a summary of the demographic characteristics of this sample. Participants were largely in the early-20s age bracket, which was only slightly above our minimum age of inclusion in the research (18 years old). For this reason, the age data were not normally distributed and the measure of central tendency for this variable in Table 1 is expressed as median (interquartile range). The majority of the students at these schools were at the Bachelor’s level, and studying Western art (classical) music, although some were also in contemporary or jazz programs. Students could be studying in any musical discipline (e.g., performance, composition and theory).

**Table 1:** Demographics.

		All participants (*N* = 549)	Only those included in factor analysis (*n* = 439)	Only those included in reliability analysis (*n* = 328)
Age		21.00 (4.00)	21.00 (4.00)	21.00 (4.00)
Gender	Female	359 (65.4%)	282 (64.2%)	208 (63.4%)
	Male	178 (32.4%)	146 (33.3%)	110 (33.5%)
	Trans female	2 (0.4%)	2 (0.5%)	2 (0.6%)
	Non-binary	6 (1.1%)	6 (1.4%)	5 (1.5%)
	Missing	4 (0.7%)	3 (0.7%)	3 (0.9%)
Country	Australia	57 (10.4%)	43 (9.8%)	28 (8.5%)
	Canada	253 (46.1%)	200 (45.6%)	164 (50.0%)
	New Zealand	31 (5.6%)	24 (5.5%)	12 (3.7%)
	South Africa	43 (7.8%)	32 (7.3%)	19 (5.8%)
	United Kingdom	27 (4.9%)	24 (5.5%)	17 (5.2%)
	United States of America	138 (25.1%)	116 (26.4%)	88 (26.8%)
Instrument	Brass	108 (19.7%)	97 (22.1%)	72 (22.0%)
	Woodwind	115 (20.9%)	93 (21.2%)	71 (21.6%)
	Percussion	24 (4.4%)	18 (4.1%)	13 (4.0%)
	Upper Strings	86 (15.7%)	79 (18.0%)	61 (18.6%)
	Lower Strings	33 (6.0%)	31 (7.1%)	24 (7.3%)
	Keyboard	115 (20.9%)	98 (22.3%)	68 (20.7%)
	Voice	41 (7.5%)		
	Plucked Strings	21 (3.8%)	18 (4.1%)	15 (4.6%)
	Other	4 (0.7%)	4 (0.9%)	3 (0.9%)
	Missing	2 (0.4%)	1 (0.2%)	1 (0.3%)
Degree Program	Bachelor/undergraduate level	261 (47.5%)	216 (49.2%)	157 (47.9%)
	Masters	39 (7.1%)	32 (7.3%)	25 (7.6%)
	Doctoral/post-doctoral	3 (0.5%)	3 (0.7%)	3 (0.9%)
	Diploma or certificate program	6 (1.1%)	3 (0.7%)	2 (0.6%)
	Missing	240 (43.7%)	185 (42.1%)	141 (43.0%)

As described in the text, these data were not normally distributed. The measure of central tendency for this variable is therefore expressed as median (interquartile range).

To ensure that data from different nations could be compared to each other, we conducted a test equivalence analysis (Abubakar et al., 2013). Cronbach’s alpha was acceptable in all countries involved in the study, with the highest alpha levels among Australia (0.915), Canada (0.932), and the United States (0.917), and only slightly lower levels among New Zealand (0.809), South Africa (0.872), and the United Kingdom (0.893). These findings demonstrate that there are no important differences between the responses of participants from different nations.

### Procedure

Research procedures were reviewed and approved by the Office of Research Ethics and Integrity at the University of Alberta, which was the institutional affiliation of the first author when the study began, as well as the local research ethics board at each of the universities from which participants were recruited. Recruitment emails and social media materials included a link to the complete survey, including the MHL-Q19 and additional four questionnaires, hosted online using the Qualtrics platform.^2^ When they had completed the survey, participants could provide their email address for follow-up to assess test–retest reliability. Those who provided their email addresses were sent a new link to a survey consisting only of the MHL-Q19, which took ~5 min to complete. Participants who completed the second survey were offered a gift card for $5 Canadian or a similar value in a local currency. In the majority of cases, the gift cards were digital and two options were offered (e.g., Starbucks and Amazon). Local contacts were consulted as to the appropriateness of the incentives at each site, and if electronic gift cards were not available, arrangements were made with a suitable vendor to distribute gift cards to participants in accordance with the policies of the local research ethics board.

The second survey (MHL-Q19 only) was sent ~2 weeks after participants had responded to the first survey. This is consistent with the relevant literature (Nunnally and Bernstein, 1994; Marx et al., 2003; Bardhoshi and Erford, 2017).

### Data analysis plan

Data were cleaned and collated. Frequencies, distribution of data and ranges of scores were calculated. Frequencies of missing items per question were evaluated. Non-parametric statistical tests were used where normality of data distribution was not evident. Normality of data was assessed by inspecting histograms, skewness, and kurtosis statistics. Responses of *don’t know* on the MHL-Q19 were considered missing data and, where there were more than 20% missing responses for any participant, that participant’s responses were deemed invalid. We did not identify any outliers. We conducted an inter-item correlation analysis of responses to the MHL-Q19 to assess potential relationships between items. Internal consistency was evaluated by calculating Cronbach’s alpha. Values of Cronbach’s *α* between 0.70 and 0.95 are considered good (Terwee et al., 2007).

In regard to construct validity, correlation testing was applied to calculate correlations between the scores on the different questionnaires. A correlation lower than 0.30 was considered weak, 0.30–0.60 moderate, and higher than 0.60 as strong (Terwee et al., 2007). As responses in the MHL-Q19 are scored using a Likert-style scale, the data were considered ordinal and nonparametric correlation statistics (Spearman *rho*) were used for hypothesis testing.

Data analyses were conducted using Statistical Package for Social Sciences (IBM SPSS) 28.0.0.0.

## Results

### Preliminary analysis

Data were cleaned and checked for missing values. As with previous HLS-EU tools assessing health literacy, any response of *don’t know* was considered a missing value. The responses *very easy*, *easy*, *difficult* and *very difficult* were considered valid responses. Participants who gave fewer than 80% valid responses across the whole survey were excluded from analysis, in line with previous tool development research in health literacy (Pelikan et al., 2019). A total of 73 participants who responded to the first survey were excluded from the factor analysis for this reason, leaving 439. A total of 37 participants who responded to the second survey were excluded for the same reason, and 126 participants did not complete the follow-up survey. Valid responses to both the first and second surveys were provided by 350 participants, of whom 22 were eliminated because they reported voice as their primary instrument. As shown in Table 1, this left 328 valid responses for the test–retest reliability analysis.

Table 2 shows descriptive statistics for responses to the MHL-Q19 included in the factor analysis and hypothesis testing (column 2). A slight floor effect was evident for responses to Questions 13–17, with 17%–25% of responses in the lowest scale option. At the second administration, only questions 15 and 16 demonstrated this floor effect. Frequencies of responses to each of the questions in the MHL-Q19 are provided as Supplementary material.

**Table 2:** Descriptive statistics of responses by scale item.

	Time 1						Time 2					
	N	Missing values	Mean (SD)	Median (IQR)	Skewness	Kurtosis	N	Missing values	Mean (SD)	Median (IQR)	Skewness	Kurtosis
Question 1	426	13	2.62 (0.679)	3 (1)	0.229	−0.398	353	9	2.6 (0.712)	3 (1)	0.272	−0.425
Question 2	428	11	2.41 (0.714)	2 (1)	0.49	−0.019	356	6	2.4 (0.679)	2 (1)	0.594	0.117
Question 3	428	11	2.45 (0.774)	2 (1)	0.332	−0.305	360	2	2.58 (0.735)	3 (1)	0.268	−0.41
Question 4	427	12	2.48 (0.845)	2 (1)	0.042	−0.59	357	5	2.57 (0.807)	3 (1)	−0.031	−0.478
Question 5	429	10	2.47 (0.789)	2 (1)	0.14	−0.405	357	5	2.51 (0.752)	2 (1)	0.196	−0.337
Question 6	418	21	2.24 (0.772)	2 (1)	0.308	−0.177	353	9	2.31 (0.756)	2 (1)	0.182	−0.255
Question 7	430	9	2.54 (0.806)	3 (1)	0.032	−0.485	360	2	2.54 (0.726)	3 (1)	−0.024	−0.266
Question 8	433	6	2.41 (0.731)	2 (1)	0.178	−0.213	360	2	2.47 (0.687)	2 (1)	0.524	−0.121
Question 9	432	7	2.42 (0.752)	2 (1)	0.081	−0.308	356	6	2.43 (0.711)	2 (1)	0.202	−0.17
Question 10	430	9	2.65 (0.733)	3 (1)	−0.088	−0.267	356	6	2.74 (0.702)	3 (1)	−0.129	−0.17
Question 11	428	11	2.52 (0.738)	3 (1)	0.007	−0.297	359	3	2.56 (0.714)	3 (1)	0.126	−0.298
Question 12	435	4	2.29 (0.769)	2 (1)	0.379	−0.087	357	5	2.37 (0.728)	2 (1)	0.185	0.065
Question 13	422	17	2.31 (0.835)	2 (1)	0.04	−0.659	357	5	2.49 (0.785)	3 (1)	−0.125	−0.416
Question 14	431	8	2.18 (0.885)	2 (1)	0.376	−0.558	357	5	2.28 (0.769)	2 (1)	0.363	−0.101
Question 15	433	6	2.12 (0.858)	2 (2)	0.298	−0.654	351	11	2.2 (0.803)	2 (1)	0.33	−0.283
Question 16	430	9	2.08 (0.854)	2 (2)	0.448	−0.415	345	17	2.17 (0.741)	2 (1)	0.276	−0.13
Question 17	420	19	2.06 (0.758)	2 (0)	0.467	0.076	345	17	2.19 (0.699)	2 (1)	0.293	0.088
Question 18	415	24	2.66 (0.73)	3 (1)	−0.353	−0.027	346	16	2.83 (0.684)	3 (1)	−0.42	0.396
Question 19	413	26	2.61 (0.776)	3 (1)	0.02	−0.437	344	18	2.78 (0.762)	3 (1)	−0.161	−0.358

We also conducted an inter-item correlation analysis of MHL-Q19 responses to assess potential relationships between items. Inter-item correlation was moderate, other than for Question 19, which was weakly correlated with 14 of the 18 other items (*r* < 0.3, *p* < 0.001).

### Exploratory factor analysis

As described above, the items comprising the MHL-Q19 were developed using an adapted version of the HLS-EU Health Literacy Matrix (Figures 1, 2). This matrix provided a number of theoretically sound options for a factor solution for the present data. For this reason, Exploratory Factor Analysis was selected as the best choice of analysis for dimension reduction.

Principal axis factoring was conducted on the 19 items, using oblique rotation (direct oblimin). Oblique factor rotation was used because it would be theoretically coherent to assume that factors of health literacy are related to one another (Sørensen et al., 2012). The Kaiser–Meyer–Olkin measure verified the sampling adequacy for the analysis (KMO = 0.91) and all KMO values for individual items were >0.857, indicating that the sample size was sufficiently large for the factor analysis. Initial analysis found four factors with eigenvalues >1. However, the mean communality was 0.456, suggesting that Kaiser’s criterion of extracting factors with eigenvalues >1 may be inappropriate. The scree plot was ambiguous, with grounds for extracting one, two, or three factors. Ultimately, we chose to retain three factors because of the convergence of support from the inflection of the scree plot and the theoretical congruence of the factors extracted.

This initial solution gave us three factors that were congruent with the theoretical foundation of the tool, in that they correspond roughly to the horizontal axis of the HLS-EU Health Literacy matrix displayed in Figure 1. The pattern matrix for this solution is displayed in Table 3 and the correlation between the factors is shown in Table 4.

**Table 3:** Pattern matrix.

	Factor		
	Risk to performance health (Disease prevention)	Healthcare	Health promotion
10. Understand information about risks to your performance health	0.823		
11. Judge which risks relate to your performance health	0.758		
8. Make informed decisions to optimize your performance health	0.598		
9. Find out about risks to your performance health	0.55		
18. Understand treatment advice if you have performance health issues	0.534	0.358	
7. Judge how performance health information applies to you	0.506		
13. Understand information about healthcare for musicians	0.387		
12. Decide how to prevent performance health issues	0.371		
19. Follow treatment advice if you have performance health issues	0.334		
16. Find treatment if you have performance health issues		0.863	
15. Judge where to seek help if you have performance health issues		0.806	
17. Judge the advantages and disadvantages of different treatment options for performance health issues		0.569	
14. Judge when to seek help if you have performance health issues		0.568	
3. Understand how your physical environment contributes to performance health			0.797
4. Understand how your social environment contributes to performance health			0.654
5. Judge how performing affects your health			0.505
2. Find reliable advice for your performance health			0.473
6. Judge if information about performance health is reliable	0.309		0.397
1. Find information about healthy performance habits			0.381

Extraction Method: Principal Axis Factoring. Rotation Method: Oblimin with Kaiser Normalization. b. Rotation converged in 15 iterations.

**Table 4:** Correlations among factors.

	Risk to performance health	Healthcare	Health promotion
Risk to performance health	–		
Healthcare	0.536	–	
Health promotion	0.585	0.372	–

### Naming the factors

The factors extracted were largely in line with the theoretical constructs of Health Promotion, Disease Prevention, and Healthcare from the HLS-EU Health Literacy matrix. Closer examination of the factor loadings for each question revealed a slightly different pattern in our findings than in the design of the questionnaire. Question 18 (“Understand treatment advice if you have performance health issues”) and Questions 19 (“Follow treatment advice if you have performance health issues”) were notable in that they both loaded onto a factor with the Disease Prevention items, rather than with Healthcare as we had anticipated when developing these items.

### Hypothesis testing

We tested our hypotheses using summary scores for each of the subscales of the MHL-Q19 determined by the factor analysis. Table 5 shows the correlations between scores for each of the subscales of the MHL-Q19 and for the other four comparator tools. The data were ordinal, so Spearman’s *rho* was used as the correlation statistic. We also conducted the hypothesis testing using an overall summary score for the MHL-Q19, and this scoring structure was likewise supported by our factor analysis.

**Table 5:** Hypothesis testing.

	Performance health			Risks			Issues			All		
	*rho*	*p*	CI (95%)	*rho*	*p*	CI (95%)	*rho*	*p*	CI (95%)	*rho*	*p*	CI (95%)
HLS-EU-Q16	0.37	<0.001	[0.28, 0.46]	0.50	<0.001	[0.42, 0.57]	0.45	<0.001	[0.37, 0.53]	0.54	<0.001	[0.46, 0.61]
MPIIQM												
Interference	−0.16	0.038	[−0.32, −0.00]	−0.16	0.042	[−0.31, 0.00]	−0.15	0.067	[−0.30, 0.02]	−0.20	0.013	[−0.35, −0.04]
Intensity	−0.10	0.215	[−0.26, 0.06]	−0.16	0.051	[−0.31, 0.01]	−0.14	0.082	[−0.29, 0.02]	−0.16	0.045	[−0.31, 0.00]
RAND												
Physical	0.08	0.114	[−0.02, 0.17]	0.23	<0.001	[0.14, 0.32]	0.17	<0.001	[0.08, 0.26]	0.21	<0.001	[0.12, 0.30]
Mental	0.18	<0.001	[0.09, 0.27]	0.26	<0.001	[0.16, 0.34]	0.24	<0.001	[0.15, 0.33]	0.29	<0.001	[0.19, 0.37]
GSE	0.17	<0.001	[0.07, 0.26]	0.19	<0.001	[0.10, 0.29]	0.20	<0.001	[0.10, 0.29]	0.22	<0.001	[0.12, 0.31]

*Hypothesis 1*: was supported in that scores on the MHL-Q19 and HLS-EU-16 were significantly moderately (rather than weakly) correlated.

*Hypothesis 2*: was not supported in that scores on the MHL-Q19 were weakly but significantly negatively correlated with scores for both MPIIQM pain interference and intensity; we had predicted a moderate positive correlation with the former, and a weak positive correlation with the latter.

*Hypothesis 3*: was partially supported in that scores on the MHL-Q19 were weakly positively correlated with both the physical and mental component scores of the RAND-12; we had predicted a stronger correlation with the former than the latter.

*Hypothesis 4*: was partially supported in that scores on the MHL-Q19 were weakly but significantly correlated with scores on the GSE; we had predicted a moderate correlation between them, on the basis of the previous literature reporting a relationship between health literacy and self-efficacy.

### Reliability

Each subscale demonstrated high reliability using Cronbach’s alpha (Health Promotion, *α* = 0.802; Risks to Performance Health, *α* = 0.854, Healthcare, *α* = 0.826). When the same analyses were run using the whole scale, Question 19 demonstrated a corrected item-total correlation (*r* = 0.408, *p* < 0.01) that was considerably lower than the other items (*r* values between 0.503 and 0.67). Cronbach’s alpha for the entire scale was also high (*α* = 0.919).

Each of the subscale scores were moderately and significantly correlated between the first and second administrations of the new tool. Health Promotion (*rho* = 0.651, *p* > 0.001), Risks to Performance Health (*rho* = 0.692, *p* < 0.001), and Healthcare (*rho* = 0.629, *p* > 0.001) all demonstrated acceptable levels of test–retest reliability. Test–retest reliability of an overall summary score was higher (*rho* = 0.779, *p* > 0.001).

## Discussion

This is the first study evaluating the validity and reliability of a new instrument for measuring musicians’ health literacy. The findings support the need for the development of such a tool and show promising psychometric features with great potential for measuring this construct. Some variation in Cronbach’s alpha levels was present across the countries where participants lived; however, in some countries multiple schools participated in the study, and participation varied between schools. However, these small differences in Cronbach’s alpha do suggest a need for future research to assess the validity of the tool in different geographical and/or cultural settings.

With respect to construct validity, some of our findings were consistent with our hypotheses, while others were not. Responses to the HLS-EU-Q16 were moderately correlated with their responses to the MHL-Q19, while we had predicted a weak correlation. This indicates that musicians’ health literacy and general health literacy are related constructs; however, the overlap between the two is not strong. This finding further justifies the need for this musician-specific health literacy assessment tool as argued by Wijsman et al. (forthcoming; see footnote 1).

Responses to the GSE were less strongly correlated with responses to the MHL-Q19 than we had predicted. As described in the Introduction, the research literature suggests that health literacy and self-efficacy are related constructs. However, these conclusions have been drawn from research using tools that are designed to measure general health literacy in large populations. Our results, by contrast, indicate that self-efficacy is less important in determining post-secondary music students’ ability to access, understand, appraise, and apply health information related to music performance. This might suggest that other factors may have more influence on music students’ health literacy, and setting the expectation that they will take responsibility for their own health and well-being may be unfounded.

While we had predicted a stronger correlation between the RAND 12 physical component scores and MHL-Q19 scores than between the mental component scores and MHL-Q19 scores, correlations between both physical and mental health component scores and the MHL-Q19 scores were weak. This suggests that music students’ health literacy is weakly related to, but not entirely predicted by, their individual health status alone.

Our results did not support the hypothesis that participants scoring higher on the MPIIQM for pain and its interference with performance would also score higher for musicians’ health literacy. While the correlation was relatively weak, these participants in fact scored lower on the MHL-Q19. In other words, lower health literacy in these student musicians was associated with more pain and interference symptoms. It is possible that students who have – and possibly struggled with – performance-related health issues, may have worse health outcomes, and therefore, less confidence in being able to cope with these issues, leading to lower health literacy scores. However, research has also shown that music students do not always consider PRHIs to be health issues (Guptill et al., 2000; Waters, 2019, 2020). Therefore, when they are faced with PHRIs, they may not try to access health information at all, nor consult healthcare professionals or identify health resources to assist them in addressing these concerns. The relationship between lower scores on the MHL-Q19 and higher scores on the MPIIQM therefore may reflect a lower level of health literacy in these music students, specifically in relation to their occupational health. It is also possible that lower health literacy may lead to more PRHIs, a primary rationale for the development of the MHL-Q19 (Wijsman et al., forthcoming; see footnote 1). The relationship between pain intensity/interference and musicians’ health literacy appears to be complex and could prove to be an important focus of future research on musicians’ health.

Figure 2 shows the questions from the MHL-Q19 and their corresponding domains from the adapted matrix created during the development of this new measurement tool. Comparing this figure with Table 3, it can be seen that the results of the factor analysis are a good but not perfect fit with the theoretical design of our questionnaire. As indicated above, Q18 and Q19 mapped onto domains that were not those we had intended when we created the tool. As a result, the construct represented by this domain, which was called Disease Prevention in the HLS-EU tools, might not represent the way participants in our survey understood these particular items. Based on the grouping provided by their responses, we have proposed a new name for this domain: Risk to Performance Health. Further evaluation of the tool could include using methods such as cognitive interviews (Willis and Artino, 2013; Willis, 2018), which ask participants to complete questionnaires while describing their thought processes as they complete the tool. Such evaluation may shed more light on how musicians relate questions in these three domains to one another.

While the internal consistency of the MHL-Q19 is high, its test–retest reliability was somewhat lower than we would have expected. Streiner et al. (2008) suggest three possible explanations for low test–retest reliability: 1. The scale itself is unreliable; 2. The construct changes over time; or 3. The participants’ perception of the construct is changed by filling out the questionnaire. The latter explanation is the most likely. In retrospect, the majority of the institutions from which we recruited participants were those with which we had previous relationships, or where we had contacts; and these institutions were more likely to be sympathetic to the need for health promotion and injury prevention education, or indeed, already offered some health education to their students. It is possible, therefore, that participants were, in fact, learning about health promotion and injury prevention over the course of the 2 weeks between the first and second administration of the MHL-Q19. It is also possible that their health literacy was quite low to begin with, and that completing the questionnaire caused them to consider these issues for the first time or to take them more seriously, and thus, this changed the construct for them. For example, some of the Health Promotion items asked participants to consider what they would do if they had a PRHI. After having been prompted to consider this possibility, their awareness and confidence in accessing, understanding, appraising and applying information related to PRHI may have increased. If this is indeed the case, it would suggest that initial interventions that were focused on raising awareness of PRHI and health literacy might have an impact. However, we cannot rule out the possibility that the wording of the questions led to lower test–retest reliability. Given that the wording of MHL-Q19 items was chosen to be simple and relatable for musicians, and to resemble the items in the well-established HLS-EU-Q tools that have been used in many countries around the world (Wijsman et al., forthcoming; see footnote 1), we think that this is unlikely.

During the factor analysis, Questions 6 and 18 were found to load onto more than one factor. In addition, as mentioned above, Questions 18 and 19 loaded onto factors that were not the ones we initially assigned to them during the design of the questionnaire, namely Disease Prevention (Risks to Performance Health) rather than Healthcare. This caused us to reflect on the questions themselves. Questions 18 and 19 would be difficult for participants to answer if they had never experienced a PRHI, and both questions also introduce an “if,” thereby requiring the respondent first to decide if they had had a PRHI, and second to decide how difficult it would be to understand or follow treatment advice they might not have received. The loading of Questions 18 and 19 onto Risks to Performance Health factor rather than Healthcare suggest that our thinking as developers when we designed these items might be somewhat different than that of participants when answering these questions. Future research employing cognitive interviews with participants about their thought processes as they complete the questionnaire, as described previously, could also help us to change the wording of the questions so that participants’ responses are better aligned with our intended outcomes.

Overall, the psychometric properties of this new tool are promising. We anticipate that this tool could be used by healthcare professionals, educators, and educational programs to measure students’ health literacy at baseline (e.g., upon entry to the program or at the beginning of treatment), to determine the effectiveness of educational interventions or healthcare. It could also be used in conjunction with measures of health to further our understanding of the relationship between musicians’ health literacy and their health and well-being.

## Limitations

Limitations of this validation study include the fact that the questionnaire was administered in English only in English-speaking, primarily higher-income countries. We intend to translate this tool into other languages and test non-English versions in a variety of countries in future.

As with the development of any tool to measure a newly identified construct, it is possible that the process we followed to develop our tool from the pre-existing HLS-EU family of questionnaires resulted in a tool that did not fully address health literacy for musicians. There are other ways that the development of such a tool could progress, such as beginning the process from qualitative interviews with musicians. Other health literacy tools have been developed using such a process (Osborne et al., 2013). However, when considering the selection of a model for the development of our tool, we rejected Osborne et al.’s health literacy questionnaire because it only addressed one of four health literacy competencies (decision-making/critical thinking, evaluation, responsibility, and confidence/self-efficacy) that were suggested in the literature to be enablers of positive health outcomes for musicians (Wijsman et al., forthcoming; see footnote 1). At this time, our tool is the only one to address health literacy in musicians, and its utility in practice and research remains to be demonstrated in future research.

During the development of the questionnaire, the MHLC chose to maintain the scoring structure of the HLS-EU including the four valid responses (*very difficult*, *difficult*, *easy*, and *very easy*), the optional response *don’t know*, scored as missing data, and the determination that 20% or more *don’t know* responses rendered the participant’s responses invalid. This decision was made to maximize the comparability with the HLS-EU suite of tools. It should be noted that the HLS-EU tools were initially designed to be administered verbally, either in person or over the telephone. It seems reasonable to assume that *don’t know* might not be selected as often when the questionnaire is administered verbally as when *don’t know* appears as an available response option in print or online, and this may explain the lower invalidity rate found for HLS-EU questionnaires than for the MHL-Q19. The developers of the HLS-EU questionnaires did, however, intend their tools to be delivered online in future applications, although the literature on the HLS-EU-Q (Sørensen et al., 2013, 2015; Pelikan et al., 2019) does not include any visual representation of how it would appear. Thus, while we acknowledge that there might be differences between the rates of invalid responses if the MHL-Q19 were to be administered verbally as well as online, we think it unlikely that these differences would be significant.

## Conclusion

In this paper, we have evaluated the validity and reliability of a new health literacy questionnaire for musicians, the MHL-Q19. The tool showed acceptable reliability. Questions that arose from the reliability evaluation point to the intriguing possibility that music students’ health literacy may be changed by completing this questionnaire, which should be explored in future research. Factor analysis indicates that MHL-Q19 questions generally map onto the domains of the health literacy conceptual framework as intended, with a few exceptions. Our hypotheses about how the new tool would perform in comparison with other validated instruments were partially supported, with some unexpected results. These can be partly explained by reflection on additional literature, and some of which point to the need for more research to further investigate the utility of this new questionnaire. In addition, because musicians’ health literacy is a developing construct, the partial support for the hypotheses described above may provide useful insight into the construct of musicians’ health literacy itself.

In summary, given that this is a new instrument, the validity evidence presented is positive and promising. Further studies are needed to refine the tool. The results from this study support the view of the MHLC that musicians’ health literacy is a distinct construct that cannot be fully evaluated with existing health literacy tools. The MHL-Q19 has demonstrated great potential for measuring this construct and we anticipate that future research will further strengthen both the tool itself and our understanding of musicians’ health literacy.

## Data availability statement

The data in this article are potentially sensitive health information. We do not have ethics approval to share them outside of the research team. Requests to access the datasets should be directed to cguptill@uottawa.ca.

## Ethics statement

The studies involving human participants were reviewed and approved by University of Alberta Research Ethics Office. A letter of information was presented as the first page of the online surveys and consent was inferred when participants completed the questionnaires.

## Author contributions

CG was the lead researcher and primary author for the manuscript, with significant contributions from TS and SW. CG, TS, VB, MRR, and SW were the primary researchers designing and implementing this study. TS, with assistance from MRR, analyzed the data. RL, BR-S, JG, and SW assisted in obtaining research ethics board approvals and recruiting participants. CG, VB, RL, BR-S, BA, JG, PV, and SW participated in the design of the MHL-Q19 questionnaire. SW is the Lead Academic for the Musicians’ Health Literacy Consortium. All authors contributed to the article and approved the submitted version.

## Funding

This study was funded by an Insight Development Grant from the Social Sciences and Humanities Research Council of Canada, 430-2018-00469. The Musicians’ Health Literacy Consortium and the development of the MHL-Q19 was made possible by the generous support of a Worldwide Universities Network (WUN) Research Development Fund grant (2018), A University of Western Australia Research Collaboration Award (2017, RA/1/1200/857) and contributions from the researchers’ respective institutions.

## Conflict of interest

The authors declare that the research was conducted in the absence of any commercial or financial relationships that could be construed as a potential conflict of interest.

## Publisher’s note

All claims expressed in this article are solely those of the authors and do not necessarily represent those of their affiliated organizations, or those of the publisher, the editors and the reviewers. Any product that may be evaluated in this article, or claim that may be made by its manufacturer, is not guaranteed or endorsed by the publisher.

## Footnotes

^1^Wijsman, S., Baadjou, V., de Lisle, R., Ginsborg, J., Guptill, C., Rennie-Salonen, B., et al. Development of the Musicians’ Health Literacy Questionnaire (MHL-Q19) (forthcoming).

^2^ www.qualtrics.com

## References

[ref01] Abubakar A. Dimitrova R. Adams B. Jordanov V. Stefenel D. (2013). Procedures for translating and evaluating equivalence of questionnaires for use in cross-cultural studies. Bull. Transilv. Univ. Braşov. Ser. VII Soc. Sci. Law 6:55.

[ref1] Ackermann B. Driscoll T. Kenny D. T. (2012). Musculoskeletal pain and injury in professional orchestral musicians in Australia. Med. Probl. Perform. Art. 27, 181–187. doi: 10.21091/mppa.2012.4034, PMID: 23247873

[ref2] Ackermann B. J. Kenny D. T. O’Brien I. Driscoll T. (2014). Sound practice—improving occupational health and safety for professional orchestral musicians in Australia. Front. Psychol. 5:973. doi: 10.3389/fpsyg.2014.00973, PMID: 25249990 PMC4158789

[ref3] Araújo L. S. Wasley D. Perkins R. Atkins L. Redding E. Ginsborg J.. (2017). Fit to perform: an investigation of higher education music students’ perceptions, attitudes, and behaviours toward health. Front. Psychol. 8, 1558. doi: 10.3389/fpsyg.2017.01558, PMID: 29066983 PMC5641399

[ref4] Baadjou V. A. Ackermann B. J. Verbunt J. A. M. C. F. van Eijsden-Besseling M. D. F. de Bie R. Smeets R. J. E. (2021). Implementation of health education interventions at Dutch music schools. Health Promot. Int. 36, 334–348. doi: 10.1093/heapro/daaa050, PMID: 32601665 PMC8049544

[ref5] Baadjou V. A. Roussel N. A. Verbunt J. A. Smeets R. J. de Bie R. A. (2016). Systematic review: risk factors for musculoskeletal disorders in musicians. Occ. Med. 66, 614–622. doi: 10.1093/occmed/kqw052, PMID: 27138935

[ref6] Baadjou V. A. Verbunt J. A. van Eijsden-Besseling M. D. F. de Bie R. A. Girard O. Twisk J. W. R.. (2018). Preventing musculoskeletal complaints in music students: a randomized controlled trial. Occ. Med. 68, 469–477. doi: 10.1093/occmed/kqy105, PMID: 30085148

[ref7] Baadjou V. A. Wijsman S. I. Ginsborg J. Guptill C. de Lisle R. Rennie-Salonen B.. (2019). Health education literacy and accessibility for musicians: a global approach. Report from the worldwide universities network project. Med. Probl. Perform. Art. 34, 105–107. doi: 10.21091/mppa.2019.2011, PMID: 31152654

[ref8] Bardhoshi G. Erford B. T. (2017). Processes and procedures for estimating score reliability and precision. Meas. Eval. Couns. Dev. 50, 256–263. doi: 10.1080/07481756.2017.1388680

[ref04] Bas-Sarmiento P. Poza-Méndez M. Fernández-Gutiérrez M. González-Caballero J. L. Falcón R. M. (2020). Psychometric Assessment of the European Health Literacy Survey Questionnaire (HLS-EU-Q16) for Arabic/French-Speaking Migrants in Southern Europe. Int. J. Environ. Res. Public Health 17:8181. doi: 10.3390/ijerph17218181 PMC7663905 33167475

[ref9] Beasley L. Kiser R. Hoffman S. (2020). Mental health literacy, self-efficacy, and stigma among college students. Soc. Work Ment. Health 18, 634–650. doi: 10.1080/15332985.2020.1832643

[ref10] Berque P. (2014). *The musculoskeletal pain intensity and interference questionnaire for musicians (MPIIQM): user guide*. Available at: http://www.musicianshealth.co.uk/MPIIQMuserguide.pdf 10.21091/mppa.2016.2015 27281378

[ref11] Berque P. Gray H. McFadyen A. (2014). Development and psychometric evaluation of the musculoskeletal pain intensity and interference questionnaire for professional orchestra musicians. Man. Ther. 19, 575–588. doi: 10.1016/j.math.2014.05.015, PMID: 24984929

[ref12] Bragge P. Bialocerkowski A. McMeeken J. (2006). A systematic review of prevalence and risk factors associated with playing-related musculoskeletal disorders in pianists. Occ. Med. 56, 28–38. doi: 10.1093/occmed/kqi177, PMID: 16275655

[ref13] Chan C. Ackermann B. (2014). Evidence-informed physical therapy management of performance-related musculoskeletal disorders in musicians. Front. Psychol. 5, 706. doi: 10.3389/fpsyg.2014.00706, PMID: 25071671 PMC4086404

[ref14] Cheak-Zamora N. C. Wyrwich K. W. McBride T. D. (2009). Reliability and validity of the SF-12v2 in the medical expenditure panel survey. Qual. Life Res. 18, 727–735. doi: 10.1007/s11136-009-9483-1, PMID: 19424821

[ref15] Chesky K. Dawson W. J. Manchester R. A. (2006). Health promotion in schools of music: initial recommendations for schools of music. Med. Probl. Perf. Art. 21, 142–144. doi: 10.21091/mppa.2006.3027

[ref09] College Music Society (2015). Facts and figures concerning music and higher education in the United States. Available at: https://www.music.org/pdf/mihe/facts.pdf

[ref16] Daykin N. (2005). Disruption, dissonance and embodiment: creativity, health and risk in music narratives. Health 9, 67–87. doi: 10.1177/1363459305048098, PMID: 15576425

[ref010] Douglas A. (2019). Music, language and mental health: Music as epistemic necessity. J. Musicol. Res. 6, 17–50.

[ref18] Fernholz I. Mumm J. L. M. Plag J. Noeres K. Rotter G. Willich S. N.. (2019). Performance anxiety in professional musicians: a systematic review on prevalence, risk factors and clinical treatment effects. Psychol. Med. 49, 2287–2306. doi: 10.1017/S0033291719001910, PMID: 31474244

[ref19] Fishbein M. Middlestadt S. Ottati V. Straus S. Ellis A. (1988). Medical problems among ICSOM musicians: overview of a national survey. Med. Probl. Perf. Art. 3, 1–8.

[ref20] Geboers B. Reijneveld S. A. Koot J. de Winter A. F. (2018). Moving towards a comprehensive approach for health literacy interventions: the development of a health literacy intervention model. Int. J. Environ. Res. 15, 1268. doi: 10.3390/ijerph15061268, PMID: 29914065 PMC6025600

[ref21] Ginsborg J. Kreutz G. Thomas M. Williamon A. (2009). Healthy behaviours in music and non-music performance students. Health Educ. 109, 242–258. doi: 10.1108/09654280910955575

[ref22] Ginsborg J. Spahn C. Williamon A. (2012). “Health promotion in higher education,” in Music, Health, and Wellbeing. eds. MacDonald R. Kreutz G. Mitchell L. (Oxford: Oxford University Press), 356–366.

[ref02] Global Access Partners (2011). Tertiary music education in Australia: task force report. Available at: https://www.yumpu.com/en/document/read/32269326/tertiary-music-education-in-australia-the-university-of-sydney

[ref23] Guptill C. Zaza C. Paul S. (2000). An occupational study of physical playing-related injuries in college music students. Med. Probl. Perf. Art. 15, 86–90. doi: 10.21091/mppa.2000.2018

[ref03] Huo T. Guo Y. Shenkman E. Muller K. (2018). Assessing the reliability of the short form 12 (SF-12) health survey in adults with mental health conditions: a report from the wellness incentive and navigation (WIN) study. Health Qual. Life Outcomes 16:34. doi: 10.1186/s12955-018-0858-2 29439718 PMC5811954

[ref24] Johnson J. A. Maddigan S. L. (2004). Performance of the RAND-12 and SF-12 summary scores in type 2 diabetes. Qual. Life Res. 13, 449–456. doi: 10.1023/B:QURE.0000018494.72748.cf, PMID: 15085917

[ref25] Jørgensen M. B. Larsen A. K. (2019). “Occupational health literacy: healthy decisions at work,” in International Handbook of Health Literacy: Research, Practice and Policy Across the Lifespan. eds. Okan O. Bauer U. Levin-Zamir U. D. Pinheiro P. Sørensen K. (Bristol: Policy Press), 347–358.

[ref26] Kenny D. Ackermann B. (2015). Performance-related musculoskeletal pain, depression and music performance anxiety in professional orchestral musicians: a population study. Psychol. Music 43, 43–60. doi: 10.1177/0305735613493953

[ref27] Kenny D. Ackermann B. (2016). “Optimising physical and psychological health in performing musicians,” in Oxford Handbook of Music Psychology. eds. Hallam S. Cross I. Thaut M. (Oxford: Oxford University Press), 633–647.

[ref28] Kenny D. T. Asher A. (2017). Gender differences in mortality and morbidity patterns in popular musicians across the lifespan. Med. Probl. Perf. Art. 32, 13–19. doi: 10.21091/mppa.2017.1004, PMID: 28282474

[ref29] Kreutz G. Ginsborg J. Willamon A. (2008). Music students’ health problems and health-promoting behaviours. Med. Probl. Perf. Art. 23, 3–11. doi: 10.21091/mppa.2008.1002

[ref05] Lamontagne V. Bélanger C. (2012). Development and validation of a questionnaire on musculoskeletal pain in musicians. Med. Probl. Perform. Art. 27, 37–42. 22543321

[ref006] Matei R. Broad S. Goldbart J. Ginsborg J. (2018). Health education for musiciansps. Front. psychol. 16:1137. doi: 10.3389/fpsyg.2018.01137 PMC6055059 30061850

[ref06] Mani S. Sharma S. Omar B. Paungmali A. Joseph L. (2017). Validity and reliability of Internet-based physiotherapy assessment for musculoskeletal disorders: a systematic review. J. Telemed Telecare 23, 379–391. doi: 10.1177/1357633X16642369 27036879

[ref30] Marx R. G. Menezes A. Horovitz L. Jones E. C. Warren R. F. (2003). A comparison of two time intervals for test-retest reliability of health status instruments. J. Clin. Epidemiol. 56, 730–735. doi: 10.1016/S0895-4356(03)00084-2, PMID: 12954464

[ref31] McKenna V. B. Sixsmith J. Barry M. M. (2017). The relevance of context in understanding health literacy skills: findings from a qualitative study. Health Expect. 20, 1049–1060. doi: 10.1111/hex.12547, PMID: 28402013 PMC5600250

[ref07] Niedorys B. Chrzan-Rodak A. Ślusarska B. (2020). Health Literacy: a review of research using the European Health Literacy Questionnaire (HLS-EU-Q16) in 2010-2018. Pielęgniarstwo XXI wieku [Nursing in the 21st Century], 19, 29–35.

[ref32] Norton N. (2016). Health promotion for musicians: engaging with instrumental and vocal teachers. Arts Humanit. High Educ. Available at: http://www.artsandhumanities.org/health-promotion-for-musicians-engaging-with-instrumental-and-vocal-teachers/

[ref33] Norton N. (2020). Considering musicians’ health and wellness literature through the lens of the behaviour change wheel. J. Music Health Wellbeing Available at: https://storage.googleapis.com/wzukusers/user-20563976/documents/019d3c23ae59443bb750806623d0ff88/Naomi%20Norton%20v2%20(1).pdf

[ref08] Nolasco A. Barona C. Tamayo-Fonseca N. Irles M. A. Más R. Tuells J.. (2020). Health literacy: Psychometric behaviour of the HLS-EU-Q16 questionnaire. Gac. Sanit. 34, 399–402. doi: 10.1016/j.gaceta.2018.08.006 30473252

[ref34] Nunnally J. Bernstein I. H. (1994). Psychometric Theory (3rd ed.). New York, NY: McGraw-Hill.

[ref35] Nutbeam D. (1998). Health promotion glossary. Health Promot. Int. 13, 349–364. doi: 10.1093/heapro/13.4.349

[ref36] Okan O. Bauer U. Levin-Zamir D. Pinheiro P. Sørensen K. (eds.). (2019). International Handbook of Health Literacy: Research, Practice and Policy Across the Lifespan. Bristol: Policy Press.

[ref37] Osborne R. H. Batterham R. W. Elsworth G. R. Hawkins M. Buchbinder R. (2013). The grounded psychometric development and initial validation of the health literacy questionnaire (HLQ). BMC Public Health 13, 658. doi: 10.1186/1471-2458-13-658, PMID: 23855504 PMC3718659

[ref38] Paarup H. M. Baelum J. Holm J. W. Manniche C. Wedderkopp N. (2011). Prevalence and consequences of musculoskeletal symptoms in symphony orchestra musicians vary by gender: a cross-sectional study. BMC Musculoskelet. Disord. 12, 223. doi: 10.1186/1471-2474-12-223, PMID: 21978278 PMC3221643

[ref39] Pelikan J. M. Ganahl K. (2017). “Measuring health literacy in general populations: primary findings from the HLS-EU consortium’s health literacy assessment effort,” in Health Literacy: New Directions in Research, Theory and Practice. eds. Logan R. A. Siegel E. R. (Clifton, VA: IOS Press), 34–59. doi: 10.3233/978-1-61499-790-0-34 28972508

[ref40] Pelikan J. M. Ganahl K. Van den Broucke S. Sørensen K. (2019). “Measuring health literacy in Europe: introducing the European health literacy survey questionnaire (HLS-EU-Q),” in International Handbook of Health Literacy: Research, Practice and Policy Across the Lifespan. eds. Okan O. Bauer U. Levin-Zamir U. D. Pinheiro P. Sørensen K. (Bristol: Policy Press), 115–138.

[ref41] Perkins R. Reid H. Araújo L. S. Clark T. Williamon A. (2017). Perceived enablers and barriers to optimal health among music students: a qualitative study in the music conservatoire setting. Front. Psychol. 8:968. doi: 10.3389/fpsyg.2017.00968, PMID: 28701968 PMC5487403

[ref42] Rauscher K. J. Myers D. J. (2014). Occupational health literacy and work-related injury among U.S. adolescents. Int. J. Inj. Control Saf. Promot. 21, 81–89. doi: 10.1080/17457300.2013.792288, PMID: 23679156

[ref43] Salonen B.L. (2018). Tertiary music Students’ Experiences of an Occupational Health course Incorporating the Body Mapping Approach. [PhD dissertation]. Bloemfontein: University of the Free State. Available at: https://scholar.ufs.ac.za/handle/11660/9652

[ref44] Schmidt B.M. (2017). Psychometric Evaluation of the Musculoskeletal Pain Questionnaire for Musicians and the Musculoskeletal Pain Intensity and Interference Questionnaire for Musicians. [master’s thesis]. Fargo, ND: North Dakota State University of Agriculture and Applied Science. Available at: https://library.ndsu.edu/ir/

[ref011] Scholz U. Doña B. G. Sud S. Schwarzer R. (2002). Is general self-efficacy a universal construct? Psychometric findings from 25 countries. Eur. J. Psychol. Assess 18, 242–251. doi: 10.1027/1015-5759.18.3.242

[ref45] Schwarzer R. (2014). *Everything you wanted to know about the general self-efficacy scale but were afraid to ask*. Available at: http://userpage.fu-berlin.de/~health/faq_gse.pdf

[ref012] Schwarzer R. Born A. (1997). Optimistic self-beliefs: assessment of general perceived self-efficacy in thirteen cultures. World Psychology 3, 177–190.

[ref46] Schwarzer R. Jerusalem M. (1995). “Generalized self-efficacy scale,” in Measures in Health Psychology: A User’s Portfolio. Causal and Control Beliefs. eds. Weinman J. Wright S. Johnston M. (Windsor: NFER-NELSON), 3537.

[ref013] Schwarzer R. Mueller J. Greenglass E. (1999). Assessment of perceived general self-efficacy on the Internet: data collection in cyberspace. Anxiety Stress Coping 12, 145–161.

[ref47] Shannon H. A. Parker A. W. (2020). Evaluation of a health literacy instrument designed for the mining industry. Health Lit Res Pract 4, e84–e93. doi: 10.3928/24748307-20200316-01, PMID: 32293688 PMC7156259

[ref48] Sørensen K. Levin-Zamir D. Duong T. V. Okan O. Brasil V. V. Nutbeam D. (2021). Building health literacy system capacity: a framework for health literate systems. Health Promot. Int. 36, i13–i23. doi: 10.1093/heapro/daab153, PMID: 34897445 PMC8672927

[ref49] Sørensen K. Pelikan J. M. Röthlin F. Ganahl K. Slonska Z. Doyle G.. (2015). Health literacy in Europe: comparative results of the European health literacy survey (HLS-EU). Eur. J. Pub. Health 25, 1053–1058. doi: 10.1093/eurpub/ckv043, PMID: 25843827 PMC4668324

[ref50] Sørensen K. Van den Broucke S. Fullam J. Doyle G. Pelikan J. Slonska Z.. (2012). Health literacy and public health: a systematic review and integration of definitions and models. BMC Public Health 12, 80. doi: 10.1186/1471-2458-12-80, PMID: 22276600 PMC3292515

[ref51] Sørensen K. Van den Broucke S. Pelikan J. M. Fullam J. Doyle G. Slonska Z.. (2013). Measuring health literacy in populations: illuminating the design and development process of the European health literacy survey questionnaire (HLS-EU-Q). BMC Public Health 13, 948. doi: 10.1186/1471-2458-13-948, PMID: 24112855 PMC4016258

[ref52] Spahn C. Voltmer E. Mornell A. Nusseck M. (2017). Health status and preventive health behavior of music students during university education: merging prior results with new insights from a German multicenter study. Music. Sci. 21, 213–229. doi: 10.1177/1029864917698197

[ref53] Stanhope J. Pisaniello D. Tooher R. Weinstein P. (2019). How do we assess musicians’ musculoskeletal symptoms? A review of outcomes and tools used. Ind. Health 57, 454–494. doi: 10.2486/indhealth.2018-0065, PMID: 30555103 PMC6685794

[ref54] Steinmetz A. Scheffer I. Esmer E. Delank K. S. Peroz I. (2015). Frequency, severity and predictors of playing-related musculoskeletal pain in professional orchestral musicians in Germany. Clin. Rheumatol. 34, 965–973. doi: 10.1007/s10067-013-2470-5, PMID: 24389813

[ref55] Streiner D. L. Norman G. R. Cairney J. (2008). Health Measurement Scales: A Practical Guide to their Development and Use. 5th Edn. Oxford: Oxford University Press.

[ref56] Suthakorn W. Songkham W. Tantranont K. Srisuphan W. Sakarinkhul P. Dhatsuwan J. (2020). Scale development and validation to measure occupational health literacy among Thai informal workers. Saf. Health Work 11, 526–532. doi: 10.1016/j.shaw.2020.06.003, PMID: 33329920 PMC7728703

[ref57] Terwee C. B. Bot S. D. de Boer M. R. van der Windt D. A. Knol D. L. Dekker J.. (2007). Quality criteria were proposed for measurement properties of health status questionnaires. J. Clin. Epidemiol. 60, 34–42. doi: 10.1016/j.jclinepi.2006.03.012 17161752

[ref58] Vaag J. Glæver F. Bjerkeset O. (2014). Specific demands and resources in the career of the Norwegian freelance musician. Arts Health 6, 205–222. doi: 10.1080/17533015.2013.863789

[ref59] Waters M. (2019). Perceptions of playing-related discomfort/pain among tertiary string students: a general overview of contributing factors. Int. J. Music. Educ. 37, 226–242. doi: 10.1177/0255761419833078

[ref60] Waters M. (2020). Perceptions of playing-related discomfort/pain among tertiary string students: a general overview of contributing factors. Music. Educ. Res. 22, 257–269. doi: 10.1080/14613808.2020.1765154

[ref61] Wayment A. Wong C. Byers S. Eley R. Boyde M. Ostini R. (2020). Beyond access block: understanding the role of health literacy and self-efficacy in low-acuity emergency department patients. Ochsner J. 20, 161–169. doi: 10.31486/toj.19.0047, PMID: 32612470 PMC7310186

[ref63] Williamon A. Thompson S. (2006). Awareness and incidence of health problems among conservatoire students. Psychol. Music 34, 411–430. doi: 10.1177/0305735606067150

[ref64] Willis G.B. (2018). “Cognitive interviewing in survey design: state of the science and future directions,” in The Palgrave Handbook of Survey Research, ed. Vannette D.L. and Krosnick J. A. (Cham: Springer Nature).

[ref65] Willis G. B. Artino A. R. (2013). What do our respondents think we’re asking? Using cognitive interviewing to improve medical education surveys. J. Grad. Med. Educ. 5, 353–356. doi: 10.4300/JGME-D-13-00154.1, PMID: 24404294 PMC3771159

[ref66] World Health Organization. Division of Health Promotion, Education, and Communication (1998). *Health Promotion Glossary*. Available at: https://apps.who.int/iris/handle/10665/64546

[ref67] Xu X. Y. Leung A. Chau P. H. (2018). Health literacy, self-efficacy, and associated factors among patients with diabetes. Health Lit Res Pract 2, e67–e77. doi: 10.3928/24748307-20180313-01, PMID: 31294279 PMC6607806

[ref69] Zaza C. (1998). Playing-related musculoskeletal disorders in musicians: a systematic review of incidence and prevalence. Can. Med. Assoc. J. 158, 1019–1025. 9580730 PMC1229223

